# Congenital Glucose–Galactose Malabsorption Presenting as Hypertriglyceridemia and Medullary Nephrocalcinosis

**DOI:** 10.3390/pediatric17050090

**Published:** 2025-09-05

**Authors:** Malika Goel, Renu Suthar, Lesa Dawman

**Affiliations:** 1 Department of Pediatrics, University of California, San Francisco, CA 94158, USA; 2 Department of Pediatrics, Postgraduate Institute of Medical Education and Research (PGIMER), Chandigarh 160012, India

**Keywords:** Medullary nephrocalcinosis, congenital glucose–galactose malabsorption, hypercalcemia, hypertriglyceridemia, hypernatremia

## Abstract

A 4-month-old male child was admitted with failure to thrive, persistent osmotic diarrhea, and presence of multiple metabolic abnormalities, which included hypertriglyceridemia, hypercholesterolemia, hypercalcemia, and medullary nephrocalcinosis. He was diagnosed with congenital glucose–galactose malabsorption (CGGM). The exome analysis showed presence of pathogenic mutation in exon 8 of the *SLC5A1* gene (c875G>A, p.Cys292Tyr). This gene codes for a sodium–glucose cotransporter called SGLT1. To date, no clinical case reports have reported hypertriglyceridemia and hypercholesterolemia with CGGM. Hypercalcemia and medullary nephrocalcinosis have also been reported only in a handful of CGGM cases worldwide. Through this case, the authors attempt to highlight the uncommon manifestation of this rare disease to facilitate timely management. Although the child died due to healthcare-associated infection (HCAI), pre-natal counseling of the family was carried out for the management of future pregnancies.

## 1. Introduction

Congenital glucose–galactose malabsorption (CGGM) is a rare autosomal recessive disorder presenting in infancy [1]. The disease is due to a mutation in the solute carrier family 5-member 1 gene, also known as the *SLC5A1* gene. The gene is responsible for coding sodium/glucose cotransporter protein. The cotransporter is present in the villi of the gastrointestinal tract and actively absorbs the monosaccharides glucose, and galactose [2]. This leads to accumulation of unabsorbed glucose and galactose in the intestinal lumen. The unabsorbed carbohydrates draw in water in the lumen and lead to osmotic diarrhea. The large amounts of diarrhea lead to electrolyte abnormalities such as hypernatremia, hyperchloremia, metabolic acidosis, resulting in failure to thrive [1,3]. Since absorption of fructose is not affected by *SLC5A1*, fructose-based formulas are used to support nutrition in patients diagnosed with CGGM [4].

With only a few hundred cases of CGGM are reported worldwide, the diagnosis of this disease requires a high index of suspicion and thorough knowledge of its common as well as rare presenting features. This is especially true in areas where it has not been reported before. It is also important to recognize that, given the handful number of cases worldwide, the knowledge of clinical presentations of this disease is still evolving, and new or first-time presenting manifestations may confound the diagnosis. Piecemealing and analyzing the individual symptoms and signs becomes important in these cases. We report a case of a 4-month-old male baby with undiagnosed CGGM. He presented with complications of CGGM and a newly reported lab finding of hypertriglyceridemia and hypercholesterolemia in CGGM. The case demonstrates a relatively newer presentation of this disease and a need to keep up-to-date with the expanding knowledge of the disease in treating physicians.

## 2. Case Report

A 4-month-old male infant was admitted with a history of frequent episodes of loose stools, reduced urine output, and failure to thrive. His examination was suggestive of a severely dehydrated child with a weight, length, and head circumference of 2.2 kg (−7.59 z score), 50 cm (−7.04 z score), and 36 cm (−5 z score), respectively. Vitals at admission were a heart rate of 110 beats per minute, respiratory rate of 38 per minute, oxygen saturation of 97%, blood pressure of 98/50 mm Hg, and a capillary filling time of less than 2 s. His birth weight was 2.8 kg, and he had never been able to regain his birth weight. He was born at full term to a non-consanguineous couple with an uncomplicated antenatal period. He was first admitted at 48 h of life, with multiple episodes of loose stools, neonatal jaundice, and hypernatremic dehydration. This was treated with intravenous rehydration and phototherapy, and he was discharged home on day 14 of life. At home, he was exclusively breast-fed but continued to have three–five watery stools per day. The stools did not contain any blood and sometimes looked watery like urine, as per the parents’ observation. The family history was significant for an elder male sibling of the patient had history of chronic diarrhea, hypernatremic dehydration, acute kidney injury (AKI), and bilateral medullary nephrocalcinosis (on ultrasound), who succumbed to the illness at day 50 of life.

At admission to the hospital, the patient’s lab data were suggestive of hypernatremia with a serum sodium (Na^+^) level of 174 mEq/L, a hyperchloremia with chloride (Cl^−^) level of 151 mEq/L, severe normal anion gap metabolic acidosis with a pH of 6.9, a bicarbonate (HCO3^−^) level of 6 mmol/L, and a carbon dioxide (CO^2^) level of 13 mm Hg. His renal parameters were abnormal, with a urea level of 225 mg/dL and creatinine of 3.13 mg/dL. His serum was noted to be visibly whitish in color or lipemic at the time of lab draws, and therefore, a lipid profile was performed. His lipid profile was abnormal, with an elevated level of triglycerides at 2660 mg/dL (normal value < 150 mg/dL), elevated total cholesterol at 384 mg/dL (normal value < 153 mg/dL), a HDL of 61 mg/dL, and an LDL of 41 mg/dL. He also had hypercalcemia, with a total serum calcium (Ca^+2^) level of 13.5 mg/dL and ionized calcium (iCal) level of 2.2 mEq/L. For the work-up of hypercalcemia, his parathormone and vitamin D levels were measured, which were 5.6 pg/mL (normal range: 15–65 pg/mL) and 9.9 nmol/L (normal range: 11–42 nmol/L), respectively. Blood and stool culture showed no bacterial growth. As a part of the work-up of hypertriglyceridemia, initially, the parents’ lipid profile was measured, which was in the normal range. As there was suspicion of a congenital osmotic diarrhea (as explained in the section on differential diagnosis), his feeds were withheld on day 1 of admission itself, and management of hypernatremic dehydration was carried out. Clinically, his urine output was low at admission, which did not improve with hydration, and he became anuric by day 3 of admission. Table 1 shows a summary of the investigations according to the day of hospitalization.

As seen in Table 1, some lab parameters, like electrolyte levels and lipid profile, started to improve gradually. By day 4 of hospital stay, his triglycerides, cholesterol, and sodium improved to 234 mg/dL, 115 mg/dL, and 153 mEq/L, respectively. However, renal parameters continued to be abnormal, and he was persistently anuric from day 3 of hospital stay. Loose stools had improved on stopping oral feeds, pointing again towards an osmotic component to the diarrhea. An abdominal ultrasound was carried out to assess the cause of his abnormal renal function, revealing bilateral small kidneys and bilateral medullary nephrocalcinosis.

Clinical exome sequencing was conducted, which showed a pathogenic homozygous missense variation in exon 8 of the *SLC5A1* gene (c875G>A), resulting in a substitution of amino acid tyrosine for cysteine at codon 292 (p.Cys292Tyr; ENST00000266088.9). This variation is previously reported as pathogenic in congenital glucose–galactose malabsorption (CGGM). The final diagnosis of CGGM was considered after the results of genetic testing.

The patient was managed with intravenous (IV) fluids, rehydration, and antibiotics. He was managed for hypernatremic dehydration with strict sodium monitoring. Due to the possibility of congenital diarrhea, suspected on the basis of persistent loose stools from birth, electrolyte abnormalities, family history, and failure to gain weight on exclusive breast milk feeding, his feeds were withheld. He had slow resolution of hypernatremia along with labs showing improvement in both hypercalcemia and hypertriglyceridemia. However, acute kidney injury (AKI) persisted, with declining urine output, along with developing features of fluid overload. To manage the developing AKI, the patient was started on peritoneal dialysis. The patient’s clinical course was complicated by healthcare-associated infection (HCAI), leading to respiratory failure and refractory shock. He was managed using the appropriate antibiotics, vasopressor therapy, and ventilation. His hemodynamic and ventilatory parameters continued to worsen, leading to his death. Parental carrier testing and pre-natal diagnosis for the next pregnancy were advised.

### Differential Diagnosis

The patient’s symptoms, family history, and lab work-up were quite unique. The ppossibility of congenital diarrhea was kept given the persistent diarrhea beginning in the neonatal period, electrolyte abnormalities at admission, positive family history, and failure to gain weight on breast milk. The control of diarrhea by stopping the feeds also pointed towards a diagnosis of congenital osmotic diarrhea. However, the presence of hypertriglyceridemia, hypercalcemia, and nephrocalcinosis complicated the picture, and other differential diagnoses were also considered. Renal tubular acidosis (RTA) was also kept as a strong possibility due to the presence of normal anion gap metabolic acidosis, failure to gain weight, and medullary nephrocalcinosis. Congenital hyperlipidemia was the third differential kept initially, but the resolution of hypertriglyceridemia and hypercholesterolemia during hospital stay, and the normal lipid profile of the parents did not support this diagnosis. Although medullary nephrocalcinosis and failure to thrive are present in RTA, hypernatremia and hypercalcemia are often not a presenting feature. As such, congenital diarrhea was the strongest differential on our list. Although we could not gather enough stool samples for lab testing, in view of a strong clinical suspicion and presence of a homozygous pathogenic variant in the SLC5A1 gene in the exome sequencing the genetic testing) final diagnosis of CGGM was made. Till these results, the child was managed symptomatically along the lines of congenital diarrhea by withholding feeds, fluid resuscitation, and symptomatic management.

## 3. Materials and Methods

This is a case report from a single center. The case report, investigations, and hospital course are for a single patient. As this is a case report involving one patient, institutional review board clearance was not required as per our institute policy. Consent from the patient’s parents was obtained and can be produced upon appropriate demand.

## 4. Discussion

Diarrheal diseases in a neonate can be classified as either acquired or congenital. Diarrheal diseases that begin in the immediate postnatal period, require critical care intervention, and are persistent are more likely due to congenital diarrheas or enteropathies (CODEs) [5]. The volume of diarrhea can be so high that it can sometimes be confused with urine, which can confound the diagnosis. In resource-limited settings, infectious causes with secondary mucosal damage and malnutrition should always be considered and ruled out by a trial of antibiotics and stool culture.

CGGM is one such CODE, which occurs due to a mutation in the *SLC5A1* gene encoding for sodium–glucose cotransporter (SGLT1). SGLT1 is a solute carrier protein coupling sodium and glucose/galactose transportation from jejunal lumen to the inside of jejunal epithelial cells [3]. These mutations result in a potentially fatal disease, presenting as intractable osmotic diarrhea in infancy, often with severe metabolic acidosis, hypernatremia, and failure to thrive, along with presence of reducing sugars in stool analysis and positive hydrogen breath test upon glucose or galactose loading. The diagnosis is confirmed by genetic analysis, which reveals a mutation in the *SLC5A1* gene. Our patient had a mutation in exon 8 of this gene (c875G>A, p.Cys292Tyr). This variant has a detrimental effect on the localization and function of SGLT1 protein and has been described previously in some parts of the world [6,7], confirming it to be a known pathogenic variant. However, this mutation has rarely been described in our country. This mutation is even more rare in children born out of non-consanguineous marriage, which posed a diagnostic challenge in our patient.

Several diagnostic challenges were faced when evaluating the index child. First was the presence of hypertriglyceridemia and hypercholesterolemia. As per our literature review, this has not been reported as a feature of CGGM. The abnormal lipid profile resolved over a period of 4 days. Due to this, a possibility of hypernatremia-induced hypertriglyceridemia was considered, as has been described in some case reports [8]. A possible explanation is the increased hepatic secretion of triglycerides, which is caused by the inhibition of lipoprotein lipase by chronic hypernatremia [8,9]. We propose that hypertriglyceridemia and hypercholesterolemia should always be evaluated in CGGM patients, as they could also have long-term cardiac implications.

Second, hypercalcemia and nephrocalcinosis are other relatively rare manifestations of CGGM. As per literature review, either of them has been reported in 20 out of 107 cases [1]. Although not fully understood, a possible hypothesis of hypercalcemia is an upregulation of epithelial calcium channels (TRPV6) in the intestine and kidney, and the active form of vitamin D (1,25-dihydroxyvitamin D3) [1,10]. This leads to hypercalcemia, which can lead to nephrocalcinosis. Another reason for nephrocalcinosis includes proximal renal tubular acidosis [11]. The nephrocalcinosis could potentially also explain acute kidney disease, as seen in this patient.

The management of these children is often difficult, and the complete elimination of galactose and glucose from the diet is the mainstay of treatment. Carbohydrate-free formulas, like Ross carbohydrate-free formula (Abbott) and 3232A (Mean Johnson), can be used. Fructose must be added as a carbohydrate source to these formulas [12]. As mentioned above, fructose is not absorbed by SGLT1 and is passively absorbed; therefore, it can serve as the carbohydrate source. Another option is using fructose-based formulas directly, for example, Galactomin 19 (not available globally) [13]. Once the child is 4 s older the gradual introduction of carbohydrates can be tried based on tolerance. Other areas of potential research for treatment can be gene transfers for *SLC5A1.* Although this area has not been explored yet, there are ongoing efforts to identify solute carrier proteins (SLCs) as potential targets [14].

Normal growth and development can be achieved in children with proper nutritional management [15]. With restrictions on many food items consumed daily, such as milk and milk products, lactose, glucose, and wheat, dietary management of CGGM can pose a significant challenge for both pediatricians and nutritionists.

## 5. Conclusions

CGGM is a rare but treatable cause of persistent diarrhea in neonates and infants, and should be suspected when multiple metabolic abnormalities like hypertriglyceridemia, hypercholesterolemia, hypernatremia, hypercalcemia, and nephrocalcinosis are present. Knowledge of this disease is still evolving, and newer clinical manifestations may become apparent. With an evolving repertoire of clinical presentations of the disease, there is a possibility that the patient might have an altogether newer clinical picture at the time of diagnosis.

## Figures and Tables

**Table 1:** Lab values of the patient on admission and over the hospital stay.

	Day of Admission (Day 1)	Day 2	Day 3	Day 4
**Na (meq/L)**	174	174	162	153
**Cl (meq/L)**	151	152	136	129
**Urea (mg/dL)**	225	192	178	186
**Creat (mg/dL)**	3.13	3.17	2.77	2.7
**Triglycerides (mg/dL)**	2663		970	234
**HDL (mg/dL)**	61		11	12
**LDL (mg/dL)**	41		107	101
**Cholesterol (mg/dL)**	378		218	115

## Data Availability

No data was created in carrying out this case report.

## References

[1] Wang W., Wang L., Ma M. (2020). Literature review on congenital glucose–galactose malabsorption from 2001 to 2019. J. Paediatr. Child Health.

[2] Mergener R., Nunes M.R., Nascimento L.P.C., Muniz V.F., Graziadio C., Zen P.R.G. (2024). Congenital glucose-galactose malabsorption: A case report about cause and consequence, not exactly in this order. Glob. Pediatr..

[3] Evans L., Grasset E., Heyman M., Dumontier A.M., Beau J.P., Desjeux J.F. (1985). Congenital selective malabsorption of glucose and galactose. J. Pediatr. Gastroenterol. Nutr..

[4] Hoşnut F.Ö., Janecke A.R., Şahin G., Vogel G.F., Lafcı N.G., Bichler P., Müller T., Huber L.A., Valovka T., Aksu A.Ü. (2023). *SLC5A1* Variants in Turkish Patients with Congenital Glucose-Galactose Malabsorption. Genes.

[5] Mantoo M.R., Malik R., Das P., Yadav R., Nakra T., Chouhan P. (2021). Congenital Diarrhea and Enteropathies in Infants: Approach to Diagnosis. Indian J. Pediatr..

[6] Martín M.G., Turk E., Lostao M.P., Kerner C., Wright E.M. (1996). Defects in Na^+^/glucose cotransporter (SGLT1) trafficking and function cause glucose-galactose malabsorption. Nat. Genet..

[7] Lodoso-Torrecilla B., Perez de Nanclares G., Garin I., Calvo-Saez A., Martinez-Fernandez de Pinedo I. (2020). Glucose and galactose malabsorption: A new case in Spain. An. Pediatr. (Engl. Ed.).

[8] Crook M., Robinson R., Swaminathan R. (1995). Hypertriglyceridaemia in a child with hypernatraemia due to a hypothalamic tumour. Ann. Clin. Biochem..

[9] Ahmed F.E., Lutfi M.F. (2015). Hypertriglyceridemia in Infants and Children with Hypernatremia. Int. J. Health Sci..

[10] Pahari A., Milla P.J., van’t Hoff W.G. (2003). Neonatal nephrocalcinosis in association with glucose-galactose malabsorption. Pediatr. Nephrol..

[11] El-Naggar W., Balfe J.W., Barbar M., Taha D. (2005). Nephrocalcinosis in glucose-galactose malabsorption, association with renal tubular acidosis. Pediatr. Nephrol..

[12] Ma M., Long Q., Chen F., Zhang T., Lu M., Wang W., Chen L. (2019). Nutrition management of congenital glucose-galactose malabsorption: Case report of a Chinese infant. Medicine.

[13] Prasad B.S., Yachha S.K. (2022). Congenital Glucose-Galactose Malabsorption in a Child. Indian Pediatr..

[14] Lin L., Yee S.W., Kim R.B., Giacomini K.M. (2015). SLC transporters as therapeutic targets: Emerging opportunities. Nat. Rev. Drug Discov..

[15] Xin B., Wang H. (2011). Multiple sequence variations in *SLC5A1* gene are associated with glucose-galactose malabsorption in a large cohort of Old Order Amish. Clin. Genet..

